# Gene expression study using real-time PCR identifies an NTR gene as a major marker of resistance to benznidazole in *Trypanosoma cruzi*

**DOI:** 10.1186/1756-3305-4-169

**Published:** 2011-09-05

**Authors:** Ana M Mejía-Jaramillo, Geysson J Fernández, Lina Palacio, Omar Triana-Chávez

**Affiliations:** 1 Grupo Biología y Control de Enfermedades Infecciosas-BCEI-SIU, Instituto de Biología, Universidad de Antioquia, Medellín, Colombia

## Abstract

**Background:**

Chagas disease is a neglected illness, with limited treatments, caused by the parasite *Trypanosoma cruzi*. Two drugs are prescribed to treat the disease, nifurtimox and benznidazole, which have been previously reported to have limited efficacy and the appearance of resistance by *T. cruzi*. Acquisition of drug-resistant phenotypes is a complex physiological process based on single or multiple changes of the genes involved, probably in its mechanisms of action.

**Results:**

The differential genes expression of a sensitive *Trypanosoma cruzi *strain and its induced *in vitro *benznidazole-resistant phenotypes was studied. The stepwise increasing concentration of BZ in the parental strain generated five different resistant populations assessed by the IC_50 _ranging from 10.49 to 93.7 μM. The resistant populations maintained their phenotype when the BZ was depleted from the culture for many passages. Additionally, the benznidazole-resistant phenotypes presented a cross-resistance to nifurtimox but not to G418 sulfate. On the other hand, four of the five phenotypes resistant to different concentrations of drugs had different expression levels for the 12 genes evaluated by real-time PCR. However, in the most resistant phenotype (TcR5x), the levels of mRNA from these 12 genes and seven more were similar to the parental strain but not for NTR and OYE genes, which were down-regulated and over-expressed, respectively. The number of copies for these two genes was evaluated for the parental strain and the TcR5x phenotype, revealing that the NTR gene had lost a copy in this last phenotype. No changes were found in the enzyme activity of CPR and SOD in the most resistant population. Finally, there was no variability of genetic profiles among all the parasite populations evaluated by performing low-stringency single-specific primer PCR (LSSP-PCR) and random amplified polymorphic DNA RAPD techniques, indicating that no clonal selection or drastic genetic changes had occurred for the exposure to BZ.

**Conclusion:**

Here, we propose NTR as the major marker of the appearance of resistance to BZ.

## Background

American trypanosomiasis, or Chagas disease, is a neglected parasitic illness widely spread throughout the Americas, from the Southern United States to Argentina and Chile. *Trypanosoma cruzi *currently infects at least 7,694,500 individuals and between 60 and 80 million remain at risk of *T. cruzi *infection in endemic countries [1,2]. There is no vaccine to prevent the infection and chemotherapy is restricted to two nitroheterocyclic compounds: nifurtimox (NFX (4[(5-nitrofurfurylidene)amino]-3-methylthiomorpholine-1,1-dioxide) and benznidazole (BZ (*N*-benzyl-2-nitroimidazole-1-acetamide) [3,4].

BZ is a nitroheterocyclic compound that contains a nitro group linked to an imidazole ring. As a pro-drug, BZ undergoes activation by enzymatic activity to have cytotoxic effects within the parasite, which is catalyzed by nitroreductases (NTRs) [5]. Because there are two possible enzymes acting on it, there are two proposed hypotheses for its toxic action. The first one postulates the generation of reactive oxygen species (ROS) following a one-electron reduction caused by NTR type II enzymatic activity. Under aerobic conditions, this induces the production of superoxide anions and causes re-cycling of the drug [6]. However, it is now known that *T. cruzi *possesses both enzymatic and non-enzymatic antioxidant defenses, making it unlikely that ROS production affects the viability of the parasite, at least at the doses used to treat the disease [7-11]. The second hypothesis proposes a two-electron reduction of the drug by NTR type I. This reaction goes through a nitroso species, to a hydroxylamine derivative using NADPH as a source of electron donors. Hydroxylamine can react to produce a nitrenium cation, which induces DNA strand breaks. Moreover, the high electrophilic intermediaries may affect other molecules within the cell [12]. There are two trypanosomal enzymes with this type of activity: prostaglandin F2α synthase or old yellow enzyme (OYE), which mediates two-electron reduction in NFX under anaerobic conditions [13] and nitroreductase I (NTR). This second enzyme has already shown strong experimental evidence associated with cross-resistance to NFX and BZ [5,12,14,15]. Despite the great efforts made to understand BZ's mode of action, it is not yet completely clear; even less thoroughly studied are the initially acquired mechanisms of resistance.

Acquisition of drug-resistant phenotypes is a complex physiological process based on single or multiple changes of genes involved in its mechanisms of action [16-18]. Many studies have been based on differential gene expression analysis in high concentrations of BZ in resistant phenotypes [19-26], but the commencement of this condition remains to be understood. For this reason, the identification of genes that are differentially expressed during progression through the susceptible drug population to a resistant phenotype in *T. cruzi *populations may help to further our grasp of acquired stable resistance mechanisms, including the basis of the drug's mode of action. Additionally, it is also important to identify gene expression and/or genetic alterations as markers of either sensitivity or resistance responses, which could be useful for treatment prognosis and as potential new therapeutic targets. Therefore, the aim of this study was to examine the initial expression changes of parasites submitted to a stepwise concentration of BZ. For this reason, we chose 19 genes suggested to be involved in escaping BZ cytotoxic effects. Their RNA expression was quantified by real-time PCR (RT-qPCR). We also investigated other issues concerning drug resistance, such as the stability of BZ-resistant phenotypes without drug pressure over a long period of time as well as the cross-resistance with other nitroheterocyclic and non-nitroheterocyclic drugs.

## Materials and methods

### Reagents

BZ and NFX were purified by organic extraction from Rochagan™ tablets (Roche, Brazil) and Lampit^® ^(Bayer, El Salvador), respectively. Stock solutions were dissolved in 100% dimethyl sulfoxide (DMSO) at a final concentration of 10 mM. G418 sulfate was purchased from AMRESCO and diluted in sterile water to a final concentration of 50 mg/ml.

### Parasite cultures

*T. cruzi *epimastigotes were cultured in liver infusion tryptose (LIT) medium supplemented with 10% (v/v) heat-inactivated fetal bovine serum (FBS) at 28°C. Cultures were maintained in exponential growth by passages every 7 days [27].

### *In vitro *induction of BZ-resistant in *T. cruzi *parasites

*In vitro *resistance to BZ was induced in the susceptible discrete typing unit (DTU) I strain M.RATTUS/CO/91/GAL-61.SUC (named Gal61) using 3 × 10^7 ^epimastigotes in the logarithmic growth phase. The parasites were exposed to stepwise concentrations of BZ beginning with its initial inhibitory concentration of 50% (IC_50_) (see Results). After this procedure, resistant parasites at different concentrations were obtained until reaching a resistant population at BZ 50 μM, which corresponds to the concentration of BZ in plasma during a chemotherapy course in humans [19,28].

### MTT assays

IC_50 _of BZ, NFX and G418 in the different phenotypes was determined using an enzymatic micromethod 3-(4,5-dimethylthiazol-2-yl)-2,5-diphenyltetrazolium bromide (MTT) per triplicate in two independent experiments [29]. *T. cruzi *epimastigotes were seeded in 96-well plates (Falcon, Ref. 353072) at 1 × 10^7 ^ml^-1 ^in 200 μl of growth medium containing different concentrations of BZ, NFX or G418. After incubation at 28°C for 96 hours, 10 μl of MTT/PMS (Sigma, USA) was added to each well and the plates incubated again for 90 min more. Cell density was determined by monitoring the reduction of MTT to formazan crystals at 595 nm in an enzyme-linked immunosorbent assay (ELISA) reader (Bio-Rad). The drug concentration that inhibits parasite growth by 50% (IC_50_) was calculated by a dose-response curve using non-linear regression analysis carried out with Prism 5.0 Software (GraphPad, San Diego, CA, USA). Finally, the resistant *T. cruzi *populations were sub-cultured without BZ for 3 months and then the IC_50 _was determined as described above with the aim of quantifying the stability in BZ-resistant phenotypes.

### Study of gene expression in phenotypes resistant to different concentrations of BZ

To evaluate the genetic expression in the different phenotypes, we looked for reported genes in drug response in a variety of kinetoplastids including *Leishmania *spp., *Plasmodium *spp., *T. brucei *and *T. cruzi*. Afterward, the selected genes were considered potential markers of resistance and grouped together according to their function (Table 1).

**Table 1:** Sequences of primers used to amplified *T.cruzi *genes using qPCR

Function	Genes/Genebank ID	Size	Forward	Reverse
**Activation system**	Old Yellow enzyme (OYE)^1^/AB075599	146 bp	5'-ACTTTCGCTTGCCTATCTGC-3'	5'-GTATTTGCTGGTCTGCCTCTTC-3'
	Nitroreductase I (NTR)^1^/XM805552	195 bp	5'-GCACGTGATTGGTATGGATG-3'	5'-CTTTGTTGGGTCAAATCGCT-3'
	NADPH-cytochrome P450 reductase (P450)^1^/DQ857724	140 bp	5'-GCATACCGGTTGGACACTTT-3'	5'-GCCTTCAGGAATGATACGGA-3'
**Detoxification system of free radicals**	Trypanothione reductase (TRYT)^1^/M38051	191 bp	5'-CTCTACAAGAAGCGGGTTGC-3'	5'-CTGAGAGTGGTGCGATCAAA -3'
	Glutathionyl spermidine synthetase (GTS)^1^/XM_815753.1	125 bp	5'-ACTTCCACCGGGTCTTTTCT-3'	5'-CTGCGGCATTCATCACATAC -3'
	Trypanothione synthetase (TS)^1^/XM_805507.1	130 bp	5'-ATCCGTTGGAGGATGAAGTG-3'	5'-TAAATGTCAGACGACGCAGC-3'
	Cytosolic tryparedoxin peroxidase (cTXP)^2^/AJ012101	169 bp	5'-AAGTGGCTGGTGCTCTTCTT-3'	5'-TTGCGCTCAATGCTTGTCCA-3'
	Mitochondrial tryparedoxin peroxidase (mTXP)^2^/AJ006226.1	137 bp	5'-TGCAACACCCTGCGACTTCTTA-3'	5'-GCCCTTGTAGTCATTCAAGCTG-3'
	Superoxide dismutase-A (SOD)^1^/U90722	184 bp	5'-GTTGAGACGTGCGGTGAATA-3'	5'-GCCTTGTGGTGTTTGGTGTA-3'
**Extracellular flow of drugs system**	Multi-drug resistant gene (MDR)^1^/XM_806226.1	188 bp	5'-ATCGCTTCTATGACCCTTCCTC-3'	5'-GGCTTCCTCCACTTCTTCGT-3'
**Folate metabolism system**	pteridine reductase-1 (ptr1)^1^/AF174398	157 bp	5'-ACAGTATCGCTGTGCGTCTG-3'	5'-AGAGAGCTGCCGAGACTGAG-3'
	Methionine adenosyltransferase (MAT)^1^/XM_799937	140 bp	5'-CTACGCCGTAATGGGACACT-3'	5'-TGTTGGGCCGAGATAAGAAC-3'
	Dihydrofolate reductase-thymidylate synthase (DHFR-TS)^1^/XM_810234	164 bp	5'-GTGGCGGTAAATGGTGGACT-3'	5'-ATGGTGGTGCGGTAAATAGC-3'
	Spermidine putrescine transporter (EPT)^2^/XM_805310.1	152 bp	5'-TGAATCCACCGTTGCGGGTCT-3'	5'-CGCATTCTTGACGCTCTGCCT-3'
**Celullar metabolism system**	Histone H1 (H1)^2^/L27119.1	124 bp	5'-GCAGCAGCCAAGAAGGCTGT-3'	5'-CGACAAAGCACGGTTGAGCCA-3'
	Zinc finger protein (ZN)^2^/XM_808517	132 bp	5'-TGCAGCACGTTCCATTGTGCC-3'	5'-CGCTGCCGCCCGTAGTTTAAT -3'
	L-threonine dehydrogenase (TDH)^2^/XM_807811	166 bp	5'-TGAACCCCTCMACGGTKTACGGTGT-3'	5'-GTACATRTGAATGGCGTAGTCYGTGG-3'
	Glycoprotein gp82 (gp82)^1^/L14824	118 bp	5'-GCCAACGATAAAGGCAGTGT-3'	5'-GTGTGGAAGAAGCGGAGAAG-3'
	Cruzipain (CZP)^2^/X54414	135 bp	5'-CATCGGTGAGGTCCTCTGTT-3'	5'-CTGTGGTATGGCTGATAGCG-3'

1: Genes analyzed for all resistant phenotypes (TcR1x-TcR5x) and the parental sensitive strain

2: Genes analyzed only for the parental sensitive strain and the TcR5x resistant phenotype.

### DNA and RNA preparations *of T. cruzi*

Genomic DNA and total RNA were isolated using the GeneJET™ Purification Kits (Fermentas, USA) as described by the manufacturers. DNA and RNA integrity was analyzed by electrophoresis in 1% agarose gels in 1× TBE (89 mM Tris borate, 2 mM EDTA [pH 8.3]), stained with ethidium bromide and visualized under UV light. Nucleic acid was quantified measuring the absorbance at 260 nm using nanodrop technology. Nucleic acid purity was assessed quantifying the A260 nm/A280-nm ratio (acceptable when the ratio was > 1.8).

### cDNA preparations

A total of 10 μg total RNA was treated with 1 U DNase I (Promega) for 2 h at 37°C and then heat-inactivated at 65°C for 10 min before reverse transcription to eliminate genomic DNA (gDNA) contamination. For first-strand cDNA synthesis, the reverse transcription reaction contained 2 μg of treated RNA, 50 μM oligo d(T), 500 ng/μl hexamer primer, 5× buffer, 10 mM dNTP, 1 U Revert Aid M-MLV (Fermentas) reverse transcriptase in a final volume of 20 μl. The mixture was incubated at 42°C for 1 h. RNA without DNase I treatment was used as a control to test gDNA contamination. After first-strand synthesis, reactions were heat-inactivated at 70°C for 20 min and then diluted in nuclease- free water.

### Real-time PCR

The primers used to amplify the selected genes using RT-qPCR were designed by PrimerExpress (Applied Biosystems, Foster City, CA, USA) (Table 1). Reactions were set up in a total volume of 20 μl using 5 μl of cDNA (diluted 1:100), 10 μl SYBRGreen I master mix (Qiagen) and 1 μM each of gene-specific primer (Table 1) and performed in the Rotor-Gene-Q (Qiagen, USA) machine. The cycling conditions were: 95°C for 15 s; 45 cycles of 95°C for 15 s, 60°C for 15 s and 72°C for 15 s with a single fluorescence measurement; a final elongation step was carried out at 72°C for 10 min. Specificity of the PCR products was confirmed by analysis of the dissociation curve. The melting curve program consisted of temperatures between 60 and 95°C with a heating rate of 0.1°C/s and a continuous fluorescence measurement. Additionally, the amplicons' expected size and the absence of nonspecific products were confirmed by analysis of the real-time PCR products in 1% agarose gels in 1× TBE, stained with ethidium bromide and visualized under UV light.

### Analysis of the expression

As an initial attempt to study mRNA levels, 12 genes were analyzed in the sensitive and resistant phenotypes. Additionally, seven more genes were evaluated for the parental and TcR5x parasites. We also performed a kinetic study of mRNA expression levels for ten genes in the parental population exposed to 50 μM BZ or 0.5% (v/v) DMSO. In this assay, mRNA levels were determined for 0, 12, 24, 48 and 72 h after exposure to BZ with the aim of evaluating which genes were up- or down-regulated as a consequence of this treatment. To analyze the differential expressions, the mRNA levels obtained for each gene were compared in every resistant phenotype with respect to the sensitive parental line or with the 0 h time for the kinetic study. In all cases, we used as a reference the expression of the hypoxanthine-guanine phosphoribosyltransferase (HPGRT) gene (Genbank: L07486) and a relative quantification defined with the following formula:

$$\text{Relative Expression}=\frac{{\left(\text{Efficienc}{\text{y}}_{\text{target}}\right)}^{\Delta \text{CP target }\left(\text{average of sensitive - average resistant}\right)}}{{\left(\text{Efficienc}{\text{y}}_{\text{reference}}\right)}^{\Delta \text{CP reference }\left(\text{average of sensitive - average resistant}\right)}}$$

All relative quantification was assessed using REST software 2009, RG mode, using the pair-wise fixed randomization test with 10,000 permutations [30,31], with PCR efficiencies calculated by Rotor-Gene-Q software v.2.02.

### Number of gene copies

The NTR gene amplification and its analyses were done as described previously. The number of gene copies was determined using a relative method [31,32] and using HGPRT as a reference gene that has one copy per haploid genome in *T. cruzi *[25].

### Enzymatic activity assays

#### Total protein extracts

Fifty milliliters of epimastigote cultures in exponential growth (50 × 10^6 ^parasites/ml) were harvested at 855 *g *for 10 min at room temperature. The parasites were washed three times with PBS 1× (NaCl 8 g/l, KCl 0.2 g/l, Na_2_HPO_4 _1.44 g/l, KH_2_PO_4 _0.24 g/l, pH 7.4) and resuspended in a protease inhibitor solution (2 mM dithiothreitol, 2 mM n-aminocaproic acid, 2 mM EDTA). This was followed by shock temperature lyses for three freezing and thawing cycles. Finally, the lysed cells were centrifuged at 2,380 *g *for 20 min at 4°C. The integrity of proteins was confirmed using denaturizing gels of (SDS-PAGE) at 10% (w/v) and the protein concentration was measured by BCA assay (Pierce, USA).

SOD enzymatic activity was performed as described by Beyer et al. (1986) with slight modifications for a total extract of proteins [33]. We also tested the enzymatic activity for P-450 under the conditions published by Portal et al. (2008) with a minor adjustment [34].

### Statistical analysis

Statistical analyses were performed using the SPSS v.14.0 software package for Windows (SPSS Inc., USA), applying the one-way ANOVA test. Means were compared using the Tukey-Kramer; when the *p*-value was less than 0.05, the difference was regarded as statistically significant.

### Genetic characterization of the BZ-sensitive and BZ-resistant *T. cruzi *populations

To determine clonal selection or mutation among sensitive and resistant parasites, the genetic variability was evaluated with different molecular markers as follows:

### Amplification of *T. cruzi *kDNA 330-bp fragment using PCR

The variable region of the minicircles of kinetoplast DNA was amplified with the primers 121 (5'-AAATAATGTACGGG(T/G)GAGATGCATGA-3') and 122 (5'-GTTCGATTGGGGTTGGTGTAATATA-3'). PCR was carried out at a final volume of 50 μl containing 50 mM KCl, 10 mM Tris-HCl, 0.1% Triton X-100, 25 ng of DNA, 37 pmol of each primer, 200 μM of dNTP, 1.5 mM of MgCl_2 _and 2.5 U of Taq DNA polymerase. PCR was performed at an initial temperature of 94°C for 3 min, followed by 35 cycles at 94°C for 45 s, 63°C for 45 s, 72°C for 45 s and a final cycle at 72°C for 10 min. The amplification products for each sample were analyzed by electrophoresis in 1% agarose gel in 1× TBE, stained by ethidium bromide and visualized under UV light.

### PCR amplification of the intergenic regions of the of spliced-leader DNA genes (SL DNA)

PCR amplification was performed in 0.2-ml microcentrifuge tubes containing 25 μl of reaction mixture. Primers for amplification of the intergenic region of *T. cruzi *SL DNA genes were: 5'-GTGTCCGCCACCTCCTTCGGGCC-3' (TC1, group II-specific), 5'-CCTGCAGGCACACGTGTGTGTG-3' (TC2, group I-specific) and 5'-CCCCCCTCCCAGGCCACACTG-3' (TC, common to groups I and II). The reaction contained 25 ng of DNA, 50 mM KCl, 10 mM Tris-HCl (pH 8.0), 0.1% Triton X-100, 200 μM of each deoxynucleotide triphosphate (dNTP), 1.5 mM MgCl_2_, 12.5 pmol of each primer, and 0.625 U of Taq DNA polymerase. PCR was carried out at an initial temperature of 94°C for 3 min, followed by 27 cycles of 94°C for 30 s, 55°C for 30 s, and 72°C for 30 s, with a final extension at 72°C for 10 min. The amplification products were analyzed by electrophoresis in 1% agarose gels in 1× TBE, stained with ethidium bromide and detected by UV light.

### LSSP-PCR and RAPDS

To obtain sequence-specific gene signatures of sensitive and resistant parasites, the LSSP-PCR and RAPDs were used. For LSSP-PCR, both minicircle variable regions (kDNA) and intergenic regions of spliced-leader genes (SL) were used as markers as described by Mejia et al. (2009) [35]. RAPD analysis was achieved using a total of three primers M13-40 (5'-GTTTTCCCAGTCACGAC-3'), L15996 (5'-CTCCACCATTAGCACCCAAAGC-3') and lgt11 (5'-GACTCCTGGAGCCCG-3') in four different reactions (all primers together and separately) as described previously by Steindel et al. (1993) [36]. All of the amplification products were analyzed in 3% agarose gels stained with ethidium bromide and visualized under UV light.

## Results

### Induction of *in vitro *BZ-resistant *T. cruzi *populations

**Figure 1:**
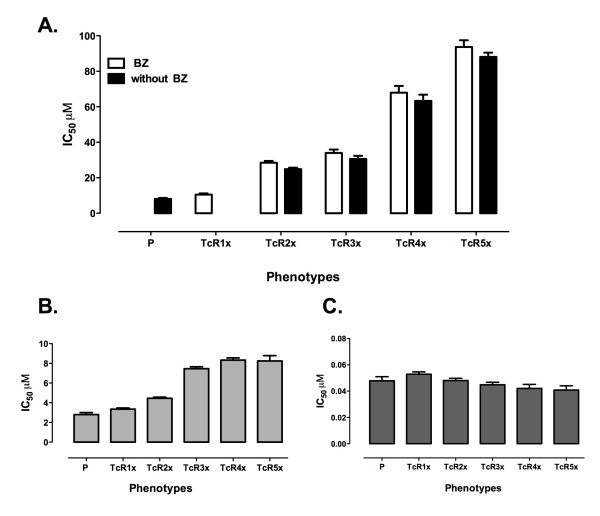
**IC_50 _to BZ, NFX and G418 in the BZ-sensitive and -resistant *T. cruzi *populations**. **A**. IC_50 _to BZ of *T. cruzi *epimastigotes of sensitive strain (P) and five resistant phenotypes (TcR1x-TcR5x) induced by stepwise concentrations of BZ; the white bars represent the IC_50 _with BZ treatment and the black bars without drug pressure over the long term. **B**. IC_50 _to NFX of P and five BZ-resistant phenotypes **C**. IC_50 _to G418 of P- and BZ-resistant lines. Statistically significant differences in IC_50 _between the parental strain and the resistant phenotypes were tested with ANOVA analysis (*p *< 0.05).

To investigate the initial mechanisms of BZ response, we used a susceptible Gal 61 *T. cruzi *as the parental strain and five different phenotypes TcR1x, TcR2x, TcR3x, TcR4x and TcR5x that were generated by *in vitro *increasing concentrations of BZ by a long-term procedure-25 passages-to reach the maximum concentration of resistance in human plasma (50 μM). We then determined the BZ doses required to inhibit 50% (IC_50_) for each available population, which ranged from 10.49 to 93.7 μM (Figure 1A, Table 2). We also found that the values obtained for the phenotypes from TcR2x to TcR5x were considered to have clearly statistically significant differences in contrast to the parental strain (*p *< 0.05) (Figure 1A). Interestingly, the resistant phenotypes also displayed a cross-resistance to NFX (Figure 1B) but did not show differences when exposed to a non-nitroheterocyclic drug, G418 sulfate (Figure 1C).

**Table 2:** BZ concentration of induction and inhibitory concentration 50% reached (IC_50_) for resistant phenotypes of Gal61

Phenotype	BZ pressure (μM)	IC_50 _BZ (μM)
Gal61	0.00	9.60
TcR1x	9.60	10.49
TcR2x	19.20	28.50
TcR3x	28.80	33.97
TcR4x	38.40	67.95
TcR5x	50.00	93.70

### Stability of BZ-resistant phenotypes

*T. cruzi*-resistant populations from TcR2x to TcR5x were grown without drug treatment for 3 months (70 generations), then the IC_50 _was determined to verify that resistant phenotypes were stable. The results did not show a statistically significant difference between the IC_50 _values for parasite growth in BZ pressure and those without drug treatment, indicating stability of BZ-resistant phenotypes over the course of time (*p *< 0.05) (Figure 1A).

### Differential gene expression in BZ-resistant phenotypes

**Figure 2:**
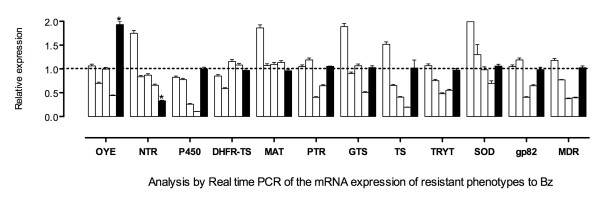
**Relative quantification using RT-qPCR of mRNA expression of 12 genes in five resistant phenotypes (TcR1x-TcR5x) compared with the sensitive strain**. The mRNA levels were normalized using the expression of the reference gene (HGPRT). The relative quantification results were obtained using the REST2009 program by randomization test with 10,000 permutations (*p *< 0.05). The graph shows the data of each gene evaluated for each of the resistant phenotypes (white bars correspond to TcR1x-TcR4x; black bars represent TcR5x phenotype) in triplicate and two independent experiments.

**Figure 3:**
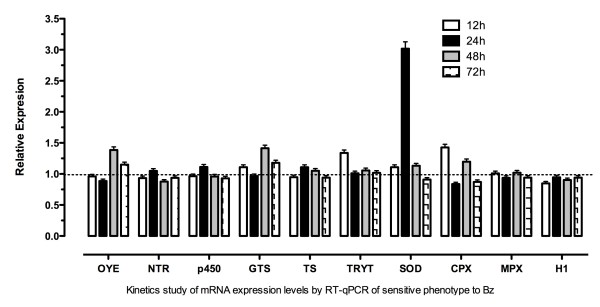
**Relative quantification by RT-qPCR of mRNA expression levels of ten genes in sensitive parasites exposed to 50 μM of BZ and evaluated at different times (12 h, 24 h, 48 h and 72 h) compared with 0 h and normalized with the expression of the reference gene (HGPRT)**. The relative quantification results were obtained using the REST2009 program by randomization test with 10,000 permutations (*p *< 0.05). The graph shows the data of each gene evaluated for each of the times in triplicate and two independent experiments.

In an initial attempt to identify differential gene expression, 12 genes were selected and their expression level was determined by RT-qPCR (Table 1). In this case, there was not a regular pattern of expression for the resistant TcR1x, TcR2x, TcR3x and TcR4x phenotypes, except for the P450, TS and MDR genes showing a gradual downregulation. However, TcR5x-resistant parasites and the sensitive strain had almost the same expression levels for all the genes evaluated, except by NTR and OYE genes that were found to be downregulated and upregulated, respectively (Figure 2). Given these results, we additionally compared the expression levels of the other seven genes involved in detoxification and other metabolic pathways between sensitive and TcR5x-resistant parasites (Table 1). Similarly, we found no differences between the two phenotypes (data not shown). To analyze the early genetic expression in response to BZ, we tested the mRNA expression levels for ten genes in a sensitive phenotype exposed to 50 μM BZ (five times its IC_50_) for different periods of time. The results indicated that there were no significant differences between the different time periods. However, some genes showed different levels of expression, especially the SOD-A gene at 24 h, but then the mRNA levels returned to the same levels of the sensitive strain at 0 h (without BZ) (Figure 3). Finally, the parasites exposed to DMSO as a control did not show changes in their mRNA levels (data not shown).

### Number of gene copies

**Figure 4:**
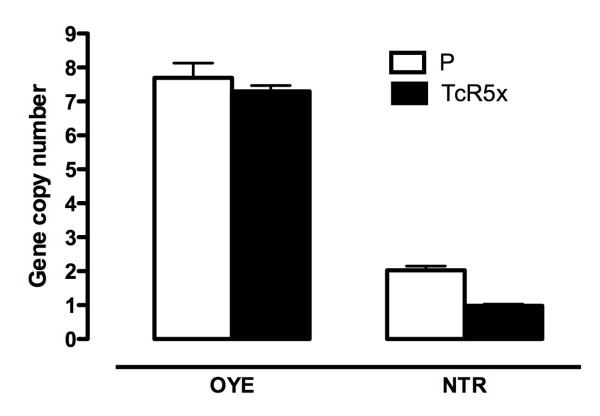
**Number of copies of OYE and NTR genes using qPCR for the sensitive parental (P) strain (white bars) and the resistant phenotype TcR5x (black bars)**. The relative quantification was normalized using the single copy gene HGPRT. The results correspond to analyses in duplicate in two independent assays.

OYE and NTR genes were found to play an important role in the therapeutic dose-resistant phenotype TcR5x, possibly decreasing the activation of the pro-drug as a first line of defense. The different regulation of transcripts for NTR and OYE genes could be due to changes in the number of gene copies. In this manner, this parameter was evaluated by qPCR. The results presented here clearly show that the number of gene copies only differed for NTR in TcR5x BZ-resistant parasites. This gene had two copies per genome diploid in the parental line, but only one was observed in the resistant phenotype (Figure 4). The OYE gene was found to have eight copies in the sensitive phenotype and it was similar for resistant parasites (Figure 4).

### Enzymatic activities for SOD and P-450

Keeping in mind that P450 is a type II nitroreductase and that SOD enzyme works on detoxification systems once the BZ has been activated, we analyzed both enzymatic activities reported to be implicated in the appearance of BZ resistance [26,34]. We found that these enzymes had similar enzymatic activities between the parental and TcR5x BZ-resistant phenotype. The specific activities of SOD for parental and TcR5x parasites were 18.708 and 18.912 U/mg, respectively, whereas P450 enzymatic activities were 8.1 × 10E-4 for the parental strain and 8.9 × 10E-4 U/mg for TcR5x parasites.

### Genetic profile of *in vitro*-induced BZ-resistant phenotypes

The drug resistance induction procedure could have been generated in the Gal61 strain clonal selection while the resistant phenotypes were being established; for this reason, the genetic profiling of resistant and susceptible parasites was studied. The genetic characterization was carried out by LSSP-PCR of kDNA and SL as well as the RAPD approach. Genetic analyses did not show evident genetic differences, which indicates a similar genetic background throughout the parasite population (data not shown). Therefore, the results showed that there was no clonal selection or even marked changes of DNA sequences from resistant parasites compared to the parental sensitive line.

## Discussion

Chagas disease is a public health problem in many tropical countries resulting from the lack of effective treatments and vaccines [37]. Two drugs are available to treat the disease, NFX and BZ, which have been previously reported as being impaired treatments with 70% of patients considered recovered in the acute stage and only 20% in the chronic stage [37]. Additionally, use of these drugs is usually followed by the appearance of parasite resistance [5]. Consequently, this study focuses on identifying markers of BZ resistance at different drug concentrations.

In recent years, many studies have been primarily concerned with the development of new treatments [38,39], with a growing tendency toward reports trying to understand how BZ and NFX work within the parasite and less toward the study of early establishment of resistance markers at different drug concentrations. For this reason, we used a high-BZ-sensitivity Gal61 strain originally isolated from a wild reservoir, and we then induced *in vitro *BZ resistance by increasing concentrations of BZ. Next, five different resistant populations were established (Table 2, Figure 1A). The BZ response is considered a complex multifactorial trait, which involves many genes of different biochemical pathways because its mode of action is not expected to implicate a specific therapeutic target [5,6]. Therefore, this study initially selected 12 genes and later seven more genes for a total of 19 genes, all of which were regarded as potential new markers of BZ-resistance phenotypes.

We found that OYE and NTR were the only two genes that showed differential expression in all the phenotypes including the TcR5x BZ-resistant population (Figure 3). Interestingly, Kubata et al. (2002) found that the OYE enzyme does not metabolize BZ, although it can metabolize naftoquinones and NFX under anaerobic conditions [13]. However, Hall et al. (2011) recently showed that the artificial overexpression of this gene in *T. brucei *did not induce NFX resistance [12]. Therefore, we believe that increased OYE levels could be a consequence of metabolic changes occurring for the down-regulation of the NTR gene without altering the resistance to BZ according to previous reports by Kubata et al. (2002) [13]. Murta et al. (2006) reported the OYE enzyme was associated with BZ resistance in induced *in vitro T. cruzi*-resistant lines due to the loss of three of its four gene copies. In this case, these authors associated its function with downstream steps after activation of the drug [25]. Here, we found that the OYE gene was overexpressed in resistant parasites. However, the BZ resistance level reached by the parasites could explain these differences in the transcription profiles found for Murta et al. (2006) and in the present study, where the maximum IC_50 _obtained was lower (50 μM). Recently, Boiani et al. (2010) found that only concentrations higher than 400 μM of NFX generated reactive oxygen species (ROS) [40]. Although this generation of ROS by BZ has not been supported by many authors [6,41], it is possible that a high concentration of the drugs has a different mode of action within the parasite, resulting in different biochemical pathways acting on it [21,26].

There have been many nitroreductase enzymes reported in several organisms, such as bacteria and protozoans, that play a role in the activation of a wide spectrum of nitro-compounds such as pro-drugs [42-45]. Members of this protein family from *Escherichia coli, Helicobacter pylori *and *Entamoeba histolytica *have shown several alterations such as frameshift mutations, decreased expression, deletions, etc., and these alterations were correlated to nitrofurazone and metronidazole resistance [46-51]. Furthermore, experimental evidence suggests that NTR is the most important enzyme in the activation of NFX, and it is also thought that *T. cruzi *uses the disruption of this enzymatic catabolism to prevent its therapeutic action through a decreased generation of toxic nitro derivatives [12,15]. Furthermore, a similar observation was made by knock-down silencing through iRNA in *T. brucei *[52]. Thus, the present results showing the downregulation of NTR are not surprising and are supported by previous findings reported by Wilkinson et al. (2008), who proposed that the loss of a single gene copy favors the development of resistance for many heterocyclic compounds in *T. cruzi *[15]. Recently, it was found that the metabolites generated through reduction of NFX by NTR in *T. brucei *induce parasite and host cell death; however, if the activation is performed by the host cell it will show no cytotoxic effects [12]. In other words, the loss of cell viability determined only by NTR activation highlights the importance of this enzyme in the mechanism of action of both nitroheterocyclic drugs, BZ as shown here and NFX as reported previously [15].

Surprisingly, the other genes evaluated did not change expression in the TcR5x phenotype, despite regulated changes in expression in the other resistant phenotypes. This could be explained as follows: the decrease of BZ activation caused by the NTR gene losing a copy in the most resistant population could prevent the formation of toxic products that altered expression of enzymes such as TS, P450 and MDR in other phenotypes. This could explain the same levels of expression found for the parental strain and the TcR5x phenotype for at least 17 other genes evaluated.

Approximately 93 genes have been reported to be involved in BZ response with different biological functions such as drug activation, transport, defense of ROS and metabolism, among others [19-23,25,26]. These genes are thought to act in molecular resistance pathways in *in vitro*-induced parasites, possibly as transitory and/or stable mechanisms to resist and survive the BZ-trypanocidal action. However, less than 5% of these genes have been confirmed in their role in the resistance mechanism and only one, the NTR gene, has been found to be directly involved in the NFX-induced resistance *T. cruzi *[12,15,52]. Interestingly, we found the same result in the BZ-induced resistance parasites.

With the aim of detecting genes with an early response to BZ and determining the possible chemotherapeutic targets of the drug, the levels of mRNA expression of the sensitive phenotype were examined when exposed to BZ at five times its IC_50 _for different periods of time. However, a specific profile of expression in the ten genes studied was not found compared to the strain without drugs. In *L. amazonensis *exposed to arsenic, major changes in the expression of proteins involved in tryparedoxin pathways were detected in the first 7 h [53]. Therefore, shorter-term assessment may be necessary to detect changes related to the genes involved in detoxification. However, it is possible that other genes that were not studied in this research are affected with relation to other pathways such as heat shock proteins.

Additionally, we investigated the specific activity of two proteins, CPR and SOD. These proteins were chosen because they could participate in the BZ's metabolism role [6], and there is some evidence of their role in resistance to BZ [26,34]. Our results indicate that enzymatic activities for both enzymes were similar among the parental strain and the TcR5x resistant phenotype in accordance with the mRNA level. Thus, recently the overexpression of two different CPRs did not confer resistance to NFX in *T. brucei *[12]. In conclusion, the level of BZ-resistant phenotypes obtained in this investigation did not support the ROS generation hypothesis.

After the down-regulated expression was found for both NTR and OYE genes, the number of gene copies was calculated using the qPCR approach. The NTR gene had two copies per diploid genome in the parental strain and just one in the TcR5x phenotype as a consequence of the loss of one copy in the acquisition of resistance to BZ, as shown in other studies of NFX conferring resistance to different nitroheterocyclic compounds [15]. These results are in accordance not only with the mRNA expression, but also with previous reports, showing that this technique is capable of detecting changes in the number of copies in an easier and less time-consuming way than other traditional techniques such as Southern blot or pulsed field electrophoresis. For the OYE gene, there were no changes in the number of gene copies, contrary to previous studies that found a loss of three gene copies out of four present in the haploid genome [25]. Therefore, it is also probable that high transcription levels are the result of physiological changes in the resistant parasites and not related to the resistant condition, as discussed above [12].

On the other hand, we found that BZ-resistance parasites presented cross-resistance to NFX (Figure 1B), based upon previous experimental evidence where it was confirmed that resistance could be shared among similar nitroheterocyclic compounds of induced and natural resistance parasites [15,54-56]. However, G418 susceptibility did not show differences within all the phenotypes (Figure 1C). In fact, the molecular mode of action of G418 is different from both NFX and BZ [57]. This means that the resistance or the susceptibility trait shares pathways between both NFX and BZ. Therefore, in the event of clinical resistance, BZ cannot be changed alternatively for NFX. As a consequence of cross-resistance among nitroheterocyclic compounds, it is necessary to search for promising new compounds.

In addition to our early drug resistance model of *T. cruzi*, we also clearly demonstrate that BZ resistance in these parasitic populations did not necessarily depend on a constant presence of the drug, which can continue over the course of time (Figure 1A). The stability of the resistant phenotype has been confirmed in many *in vitro *and *in vivo *models through different parasite species [19,54]. This condition is the result of many cases of stable genetic alterations such as mutations, amplifications and deletions [16,18,58].

Alves et al. (1994) observed alterations in the restriction fragment length polymorphism of the kDNA in *T. cruzi *submitted to a number of passages during exponential growth phase or after subcloning. This phenomenon was called transkinetoplastid [59] and it consists of fast changes in the kDNA minicircle population leading to different restriction profiles. Thus, LSSP-PCR based on the kDNA variable region can reveal not only changes in the kDNA, but also the profiles of different clones. In this manner, LSSP-PCR and RAPDs analysis have been shown to be good tools in evaluating the genetic variability within the clones belonging to the same strain. These approaches are able to identify multiple or simple variations on the DNA sequence and genetic characterization of *T. cruzi *[35,60-62]. Furthermore, the high variability of kDNA markers has been extensively used to identify many clones within a strain [63]. We induced resistance using a susceptible Gal61 strain as a parental line, which was also genetically stable through different induced BZ-resistant phenotypes using kDNA and SL genes as well as the RAPD approach. Therefore, there was no evidence of clonal selection of resistant parasites or even drastic changes in DNA sequences from resistant parasites compared to the parental sensitive line.

Finally, these results indicate that exposure to BZ leads to early irregular expression of different genes possibly involved in response to cell stress, but the loss of one copy of the NTR gene could be responsible for the acquisition and maintenance of a BZ-resistant line. However, it is necessary to study whether NTR is involved in natural resistance and to use other tools such as next generation sequencing to know whether or not there are other genes affected.

## Conclusion

Diminished mRNA levels of NTR and a loss of one copy of this gene in the BZ-resistant phenotype of *T. cruzi *were documented. Therefore, we propose that the NTR gene is involved in the emergence of resistance to BZ and could be used as a reliable marker of resistance in patients treated for *T. cruzi *infection.

## Competing interests

The authors declare that they have no competing interests.

## Authors' contributions

Conceived and designed the experiments: AMMJ, GJF, OTC. Performed the experiments: AMMJ, GJF, LP. Analyzed the data: AMMJ, GJF, LP. Wrote the paper: AMMJ, LP, OTC. All authors read and approved the final manuscript.
